# Detrended fluctuation analysis of heart rate variability during exercise: Time to reconsider the theoretical and methodological background. Comment on: Cassirame et al.`s (2025) Detrended fluctuation analysis to determine physiologic thresholds, investigation and evidence from incremental cycling test. Eur J Appl Physiol 125:523–533

**DOI:** 10.1007/s00421-025-05859-2

**Published:** 2025-07-04

**Authors:** Olaf Hoos, Thomas Gronwald

**Affiliations:** 1 Center for Sports and Physical Education, Faculty of Human Sciences, Julius-Maximilians-University Wuerzburg, Wuerzburg, Germany; 2 Institute of Interdisciplinary Exercise Science and Sports Medicine, MSH Medical School Hamburg, Hamburg, Germany; 3 G-Lab, Faculty of Applied Sport Sciences and Personality, BSP Business and Law School, Berlin, Germany

Cassirame et al.’s (2025) study on Detrended Fluctuation Analysis (DFA) to determine physiologic thresholds adds value to the recently highly debated subject of deriving exercise intensity thresholds from (non-linear) heart rate variability (HRVT). The study sheds light on relevant methodological problems and influential factors when using the short-term scaling exponent alpha1 of DFA (DFAa1) as a non-linear metric of HRV in applied exercise settings. Casssirame et al. (2025) point out that the signal-to-noise-ratio, movement artefacts as well as sex and cardiovascular fitness seem to be highly influential on the degree of agreement between the DFAa1 based threshold approach in comparison with physiological thresholds based on gas exchange (GE) data. While several most recent original studies point to similar problems and/or influencing factors when using DFAa1 for exercise intensity prescription (Gronwald et al. 2024; Sheoran et al. 2024; van Rassel et al. 2025) and an extensive review already acknowledged the wide variation in agreement depending on the chosen reference and/or HRVT method, but still suggested rather promising perspectives for this and other HRVT approaches (Kaufmann et al. 2023), Cassirame et al. (2025) generally question the use of DFAa1 (and HRVTs in general) as a method for monitoring exercise intensity using Occam’s razor as a rationale.

From our perspective, it seems valuable to use Cassirame et al.’s (2025) methodological approach and their conclusions as a starting point to take a step back and reconsider the theoretical and methodological rationale behind DFAa1 of HRV during exercise before drawing general conclusions on its value for exercise intensity prescription.

First, without a doubt careful data assessment and treatment are mandatory in HRV analysis during exercise to control signal-to-noise-ratio, artefact detection/correction and large among-subject variability and restrict external random noise as good as possible. However, these aspects are also relevant to other concepts of threshold-based exercise intensity partitioning. In addition, and most importantly, when using a *complementary* method such as DFAa1 (or other non-linear HRV approaches) during exercise, it seems necessary to consider and adhere to the underlying theoretical and methodological assumptions of that method. In this case, DFA during exercise is theoretically rooted in systems biology, or more specifically, Network Physiology of Exercise (NPE), which defines exercise regulation as a complex, goal-directed, and context-dependent dynamic mechanism in response to continuously emerging organismic and environmental demands and focuses on the interaction dynamics of complex adaptive systems (CAS) (Balagué et al. 2020). From the perspective of the NPE approach, DFAa1 of HRV during exercise could serve as a method to assess the stress-related complex neuro-autonomic regulation of an athlete, which involves several hierarchically and heterarchically organized control circuits (Persson 1996), operating on different time scales by capturing short-term correlation properties of heart rate time series. Based on the signal classification of physiological time series, DFAa1 marks the change from fractional Brownian Motion (fBM, > 1.0) to fractional Gaussian Noise (fGN, < 1.0), with 0.75 corresponding to the halfway decay within the fGN domain, which is a common marker in both dose–response modelling (Di Veroli et al. 2015) and risk stratification (Huikuri et al. 2000), and 0.5 representing the total loss of the complex interrelated organization of the cardiovascular control circuits involved (Eke et al. 2002). Taken together, DFAa1 during exercise rather demarcates a *transitional change in physiological control*. Relating these theoretical and methodological aspects to the statement by Cassirame et al. (2025) that there are no clearly identified physiological phenomena associated with DFAa1 values of 0.75 and 0.5, it seems clear that this statement is not true when placed in the context of NPE (Balagué et al. 2020).

The additional statement by Cassirame et al. (2025) that the association of biological or physiological laws with fixed numbers is always risky, using the historical concept of Mader et al.’s fixed blood lactate concentration (BLC) threshold of 4 mmol/L as an example, is also rather misleading. In contrast to the fixed value of 4 mmol/L, which had no meaning in itself, but rather served as an estimate of maximum lactate steady state using a particular protocol and type of exercise, the DFAa1 fixed values are directly related to signal classification of physiological time series of CAS. In this context, it could be argued that the calculated DFAa1 values may reveal different scaling behaviors with different exercise intensities and/or durations which might imply a dynamic modification of the method (e.g. dynamic DFA, Kanniainen et al. 2023), but the interpretation from an NPE perspective remains related to physiological signal classification and a universally designed internal calibration framework of complex system dynamics. Whether individual markers of, for example, the kinetics of DFAa1 during incremental and/or constant load exercise might have additional value remains an open question that could be addressed in future research.

Consequently, the relative proximity of DFAa1 values during incremental exercise to metabolic thresholds, which mostly represent a deviation from the linearity of GE and/or BLC-related values, is valuable because it provides a first impression of whether such a complementary method is consistent with, and could be used like exercise intensity prescriptions from metabolic thresholds that have a long tradition in results-proven training practice. However, the metabolic perspective of GE and/or BLC-based threshold concepts *does no*t represent a gold standard for an NPE-based approach that evaluates complex physiological interaction on different time scales (Balagué et al. 2020). Thus, even a *mismatch* between these approaches does not per se *disqualify* DFAa1 for exercise intensity prescription in general (Kaufmann et al. 2023). A true evaluation of exercise prescription models should rather use a longitudinal comparison of different methods (e.g. based on GE/BLC, HRV) and show which of them more easily translates from incremental exercise testing to (prolonged) endurance training in the field, provides better perspectives for day-to-day fine-tuning in different environmental settings and most importantly supports optimized training adaptations in the relevant physiological and/or performance outcomes.

Second, for DFA during exercise indeed a careful and rigorous methodological approach is mandatory, and this does not only involve the necessity for rigorous standardization and transparent reporting of the general DFAa1 settings (e.g., Kubios HRV software preferences: smoothness priors detrending: lambda = 500; scaling window DFAa1: 4 ≤ n ≤ 16; time-varying window size: 2 min, grid interval: 5 s). One possible reason for bias might also be attributed to the choice of start and end point of linear regression that should capture the rapid near-linear decline from values near 1.0 to approximately 0.5 (Rogers et al. 2021), which might have a strong influence on the slope of the regression line and thus the corresponding values of power/speed or heart rate at fixed DFAa1 values of 0.75 and 0.5. This subjective bias may also play a role in the data of Cassirame et al. (2025), because when we re-digitize the data points of Fig. 3 using professional software (PlotDigitizer, Porbital/USA) and apply the original approach of Rogers et al. (2021), we obtain a 20 s/45 s correction in time closer to the chosen reference thresholds compared to the HRVT1/HRVT2 values shown in Fig. 3. The possibly large inter-individual differences in the DFAa1 decay profiles and the already highlighted importance of DFAa1 data point selection and regression fit (van Rassel et al. 2025) further support the relevance of start and end points for regression. A possibility to reduce this inherent subjective bias might be the use of multiphasic dose–response modelling (Di Veroli et al. 2015) which for example led to highly superior goodness of fit (increased by > 25%) in our most recent study on DFAa1 in prolonged running (Gronwald et al. 2024) and uses the whole DFAa1-exercise intensity curve without the need for a selection of start and end point. In this context, further methodological attention needs to be drawn to the fact that values of the time-variant DFAa1 outcome of Kubios HRV software are automatically time-shifted with half the size of the chosen sliding window. Thus, the DFAa1 time axis, which starts at zero and directly aligns with the GE data time axis in Fig. 3 of the Cassirame et al. (2025) study, appears to be questionable.

Finally, a general notion should be made on the *beauty of simplicity* that is inherent to Occam’s razor. Although this rationale is always appealing, Occam`s razor has a very limited role in systems biology and NPE where interaction dynamics of CAS with multiple (redundant) feedforward and feedback control loops are rather the rule than the exception (Balagué et al. 2020). Here, oversimplification might even hinder scientific progress (Hickam`s dictum) as too simplistic methods and reasoning may not reveal anything about the ‘true’ regulation of CAS, which means that specific methods for systems biology are mandatory (Westerhoff et al. 2009).

In summary, the application of DFAa1 of HRV during exercise requires a rigorous methodological approach that addresses the inherent problems of signal-to-noise ratio, artifact detection/correction, and between- and within-subject variability, as well as an acknowledgement of its strong theoretical roots in systems biology and/or NPE. When used with appropriate methodology and adequate physiological rationale for CAS, the method could shed complementary light on endurance exercise monitoring *in addition* to standard metabolic approaches for both exercise (intensity) prescription and durability assessment (Gronwald et al. 2024). In this regard, DFAa1 during exercise should be further evaluated as a *complementary approach in NPE*, rather than being valued for its ease of use in approximating metabolic thresholds or discarded as not being interchangeable with these concepts. 
